# Piezotronic Sensor for Bimodal Monitoring of Achilles Tendon Behavior

**DOI:** 10.1007/s40820-025-01757-6

**Published:** 2025-04-29

**Authors:** Zihan Wang, Shenglong Wang, Boling Lan, Yue Sun, Longchao Huang, Yong Ao, Xuelan Li, Long Jin, Weiqing Yang, Weili Deng

**Affiliations:** 1 Key Laboratory of Advanced Technologies of Materials (Ministry of Education), School of Materials Science and Engineering, Southwest Jiaotong University, Chengdu, 610031 People’s Republic of China; 2 Research Institute of Frontier Science, Southwest Jiaotong University, Chengdu, 610031 People’s Republic of China

**Keywords:** Piezotronic sensor, ZnO nanorods, Y-ion doping, Bimodal detection, Achilles tendon monitoring

## Abstract

Bimodal pressure sensors capable of simultaneously detecting static and dynamic forces are essential to medical detection and bio-robotics. However, conventional pressure sensors typically integrate multiple operating mechanisms to achieve bimodal detection, leading to complex device architectures and challenges in signal decoupling. In this work, we address these limitations by leveraging the unique piezotronic effect of Y-ion-doped ZnO to develop a bimodal piezotronic sensor (BPS) with a simplified structure and enhanced sensitivity. Through a combination of finite element simulations and experimental validation, we demonstrate that the BPS can effectively monitor both dynamic and static forces, achieving an on/off ratio of 1029, a gauge factor of 23,439 and a static force response duration of up to 600 s, significantly outperforming the performance of conventional piezoelectric sensors. As a proof-of-concept, the BPS demonstrates the continuous monitoring of Achilles tendon behavior under mixed dynamic and static loading conditions. Aided by deep learning algorithms, the system achieves 96% accuracy in identifying Achilles tendon movement patterns, thus enabling warnings for dangerous movements. This work provides a viable strategy for bimodal force monitoring, highlighting its potential in wearable electronics.

**Supplementary Information:**

The online version contains supplementary material available at 10.1007/s40820-025-01757-6.

## Highlights

- The underlying mechanism governing the modulation of carrier transport by piezoelectric potential is elucidated through finite element simulations and experimental validation.
- The bimodal piezotronic sensor (BPS) exhibits exceptional responsiveness to both static and dynamic stimuli, achieving an ultrahigh gauge factor of up to 23,439.
- The BPS demonstrates robust capability for bimodal monitoring and hazard warning of Achilles tendon behavior, achieving an accuracy of 96%.

**Supplementary Information:**

The online version contains supplementary material available at 10.1007/s40820-025-01757-6.

## Graphical abstract

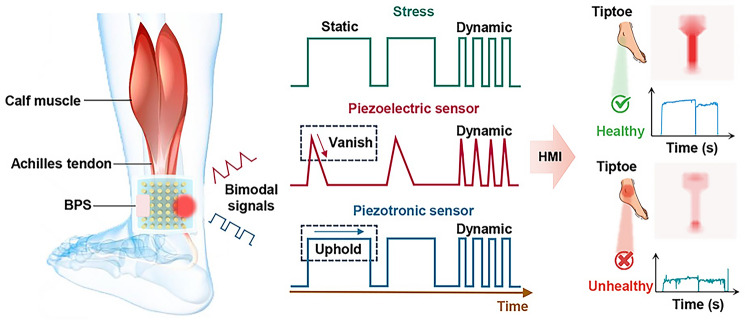

## Introduction

Understanding complex external forces has promoted significant advancements in stress sensors [1–3], enabling their crucial applications in cutting-edge areas, such as human–computer interaction, electronic skin, bio-robotics, and health monitoring [4–7]. Notably, many scenarios require the simultaneous monitoring of dynamic and static stresses, placing stringent and urgent requirements on the performance of stress sensors [8–13]. In recent years, efforts to develop bimodal sensors capable of detecting both dynamic and static stresses have mainly focused on compounding multiple functional layers to capture signals separately [14–16]. According to different working principles, conventional bimodal sensors can be primarily categorized into piezoresistive–piezoelectric, piezoresistive–triboelectric, and piezoelectric–triboelectric configurations [17–20]. However, these devices often suffer from complex structures, severe signal crosstalk, and high manufacturing costs. Therefore, achieving bimodal sensing within a single material has emerged as a key focus of current research [21–23].

Piezotronic sensors, which leverage the unique piezotronic effect, have received widespread attention due to their brand-new regulation mechanism [24–26]. By coupling piezoelectricity with semiconductor properties, piezotronic sensors could exponentially modulate the carrier transport by strain-induced piezoelectric potential at the interface between the piezoelectric material and the metal electrode. This mechanism enables the direct correlation between mechanical stimuli and changes in electrical output, resulting in excellent sensitivity to mechanical inputs [27–29]. Various materials, including ZnO, MoS_2_, and GaN, have been explored for piezotronic sensors [30–32]. A landmark achievement was the development of the first piezotronic sensor based on a transverse single ZnO nanowire [33], which utilized piezoelectrically polarized charges at the interface of two back-to-back Schottky contacts to modulate electrical transport properties, achieving a gauge factor of up to 1250. More recently, a piezoelectric tunnel junction strain sensor based on HfO_2_ and n-ZnO demonstrates remarkable performance with a gauge factor as high as 4.8 × 10^5^ and an on/off ratio of 478 at 0.10% strain [25]. Furthermore, Li ions-doped ZnO piezotronic sensor array enables large-scale integration and in-plane strain detection, demonstrating a broad application prospect [34]. As can be seen, ZnO has aroused intensive attention as a material of choice for piezotronic sensors, due to its rich properties, low cost, and ease of large-scale integration [35–37]. Despite these milestones, current research has predominantly focused on enhancing sensitivity and on/off ratios, while little attention has been paid to their possible bimodal response capabilities [38–40].

In this work, we present a bimodal piezotronic sensor (BPS) based on rare-earth (Y)-ion-doped ZnO and demonstrate its unique application in monitoring Achilles tendon behavior. Distinguished from the reported works, the BPS not only achieves high sensitivity but also exhibits good dynamic and static force detection capabilities, greatly enhancing the stability of practical implementation. Meanwhile, experimental investigations and finite element simulations are combined to probe the modulation mechanism of the effect of piezoelectricity on the performance of piezotronic sensors. As a proof-of-concept, the developed BPS demonstrates the accurate identification of different Achilles tendon states. The bimodal sensor construction scheme proposed in this work provides a new idea for bimodal detection and demonstrates broad application prospects in flexible artificial intelligence.

## Experimental Section

### Fabrication of BPS

The undoped and Y-doped ZnO nanorod films were synthesized via a low-temperature hydrothermal process. First, conductive glass (20 mm × 20 mm) underwent a thorough cleaning procedure involving sequential ultrasonication in acetone, ethanol, and deionized water, followed by nitrogen gas drying. Then, a seed layer of ZnO was deposited on the cleaned substrates using RF magnetron sputtering for 2 h. The flow rate of argon and oxygen was 40:1, and the sputtering power was 60 W. The hydrothermal growth solution was prepared by dissolving 0.1 M Zn(NO_3_)_2_⋅6H_2_O, 0.1 M C_6_H_12_N_4_ and 0.01 M Y(NO_3_)_3_⋅6H_2_O in a blue-necked bottle. The seeded substrates were then immersed in the growth solution and placed in an oven at 85° for 6 h. Subsequently, a polymethyl methacrylate (PMMA) barrier layer was uniformly coated on the surface of the ZnO films. The spin-coating speeds were 500 r min^−1^ for 10 s and 3000 r min^−1^ for 30 s, and baked at 80 °C for 1 h. For electrical characterization, a planar electrode structure was fabricated by depositing Ag electrodes (4 mm × 5 mm) through RF magnetron sputtering. The electrodes were drawn through copper wires and reinforced with silver paste. Finally, the completed device was encapsulated with commercial PU film to protect the structure during subsequent measurements.

### Characterizations

The surface morphology and the crystalline interplanar spacing of ZnO thin films were characterized utilizing transmission electron microscope (TEM, JEM-2100F) and scanning electron microscope (SEM, JSM-7800 F). X-ray diffractometer (XRD, DX-1000) was used to obtain XRD spectra. For electromechanical measurements, the pressure was applied using a linear motor (HS01-37 × 166) mounted with a digital force gauge (IMADA model ZPS-DPU-50N). The measurements of I–V characteristics were tested using a Keithley 4200 test system with a sweeping voltage between − 3 and + 3 V. During the periodic I-T characteristic test, the applied voltage was -0.6 V. The electrical performances of the samples were recorded using a Keithley 6514 system. Mott–Schottky plots were obtained from the CHI660E electrochemical station. Optical absorption spectra were acquired using a UV–visible spectrophotometer (UV2310II). The surface piezoelectric response (PR) was investigated by AFM (Bruker Multimode 8) with PFM (piezo response force microscopy) mode.

### Deep Learning for Achilles Tendon Behavior Monitoring

The convolutional neural network (CNN) models were developed in Python based on Tensor flow and Keras kernel. A specialized 1D-CNN model was developed for efficient feature extraction and automatic human motion recognition. The network was designed to process input data sequences with a fixed length of 100 data points. The dataset was strategically partitioned for model development, with 80% allocated for training, 10% for validation, and the remaining 10% for testing the corrected optimal model parameters. Model optimization was performed using a stochastic gradient descent algorithm with momentum. The performance of the model was quantitatively evaluated through classification accuracy and loss function, enabling systematic parameter tuning for optimal deep learning performance. Through iterative training and validation, the 1D-CNN model achieved robust recognition of Achilles tendon behavior patterns within 100 training epochs. Finally, the classification and analysis were carried out in terms of classification accuracy, rate, and loss function.

## Results and Discussion

### Working Mechanism of BPS and Achilles Tendon Monitoring

**Fig. 1:**
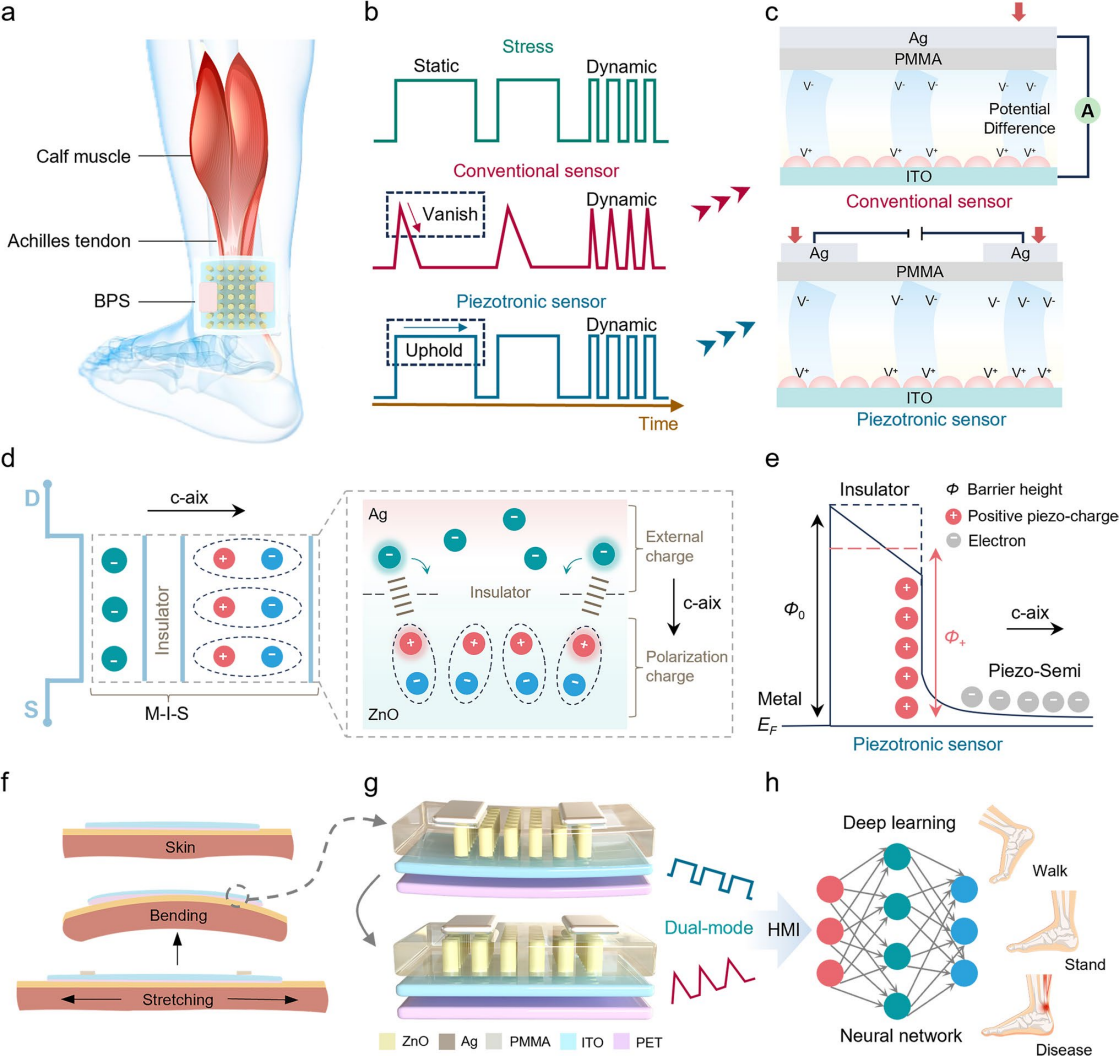
Conceptual design and working mechanism of the BPS. **a** Schematic illustration of the human Achilles tendon behavior monitoring system based on the BPS. **b** Conventional ZnO-based piezoelectric sensors are unable to detect static force due to the induced charge dissipating when an external force is maintained, while the piezotronic sensor can detect both static and dynamic forces. **c** Structural comparison between conventional ZnO-based piezoelectric (top) and piezotronic sensor (bottom). **d** Schematic diagram of the micro-working mechanism of the BPS. **e** Schematic of the metal/insulator/piezoelectric semiconductor structure of the BPS and corresponding conduction energy band profiles for the positive piezo-charge. **f** Deformation of BPS after being attached to the Achilles tendon, and **g** deformation of ZnO nanorod arrays, and **h** data processing workflow illustrating movement state analysis through deep learning

The human Achilles tendon, which connects the calf muscles to the heel bone, is vital to athletic function and daily mobility. However, it is difficult to decouple the behavior of the Achilles tendon because it often contains mixed dynamic and static forces [41–43]. Conventional methods for Achilles tendon detection primarily rely on large-scale instruments or implantable piezoelectric materials, which are associated with significant limitations, including high costs, extended testing cycles, and the inherent risk of in vivo implantation [44]. To this end, we designed a Y-doped ZnO-based bimodal piezotronic sensor for comprehensive Achilles tendon monitoring (Fig. 1a). Typically, conventional piezoelectric sensors are typically known for their good dynamic response, yet they struggle with monitoring static forces. As shown in Fig. 1b, when subjected to a constant external force, conventional piezoelectric sensors generate transient electrical signals due to charge separation, but these signals rapidly decay due to charge neutralization. In contrast, the developed piezotronic sensor demonstrates a unique bimodal response, maintaining stable electrical output that accurately tracks both changing and constant force. Taking ZnO as an example, the fundamental distinction between the conventional piezoelectric sensor and the piezotronic sensor is schematically illustrated in Fig. 1c. Conventional piezoelectric sensors employ a sandwich structure that generates potential differences across the material thickness. While the piezotronic sensors, equipped with an external power source, utilize the piezoelectric potential to modulate interfacial barriers, thereby controlling current flow through the sensor. The working mechanism of the BPS, based on a metal/insulator/piezoelectric semiconductor (M-I-S) structure, is schematically depicted in Fig. 1d. Taking an n-type semiconductor as an example, we define the silver electrode at one end as the drain and the other as the source. The device generates positive piezoelectric charges at the insulator/semiconductor interface when strained along the c-axis [45]. Specifically, this piezoelectric polarization effectively lowers the potential barrier height, facilitating electrons crossing the barrier and generating measurable current changes (Fig. 1e). Due to the external power supply, the change in the potential barrier height under a constant external force can always be maintained, thus providing the ability to detect static forces. When attached for Achilles tendon monitoring, as shown in Fig. 1f, the BPS tracks Achilles tendon deformation through corresponding bending or elongation, which in turn converts the mechanical strain into an electrical output via ZnO nanorod arrays (Fig. 1g). Subsequent deep learning analysis enables accurate determination of Achilles tendon status, movement patterns, and overall tissue health, representing a significant advancement in non-invasive biomechanical monitoring technology (Fig. 1h).

### Microstructure and Fundamental Characterization of ZnO and Y-ZnO

**Fig. 2:**
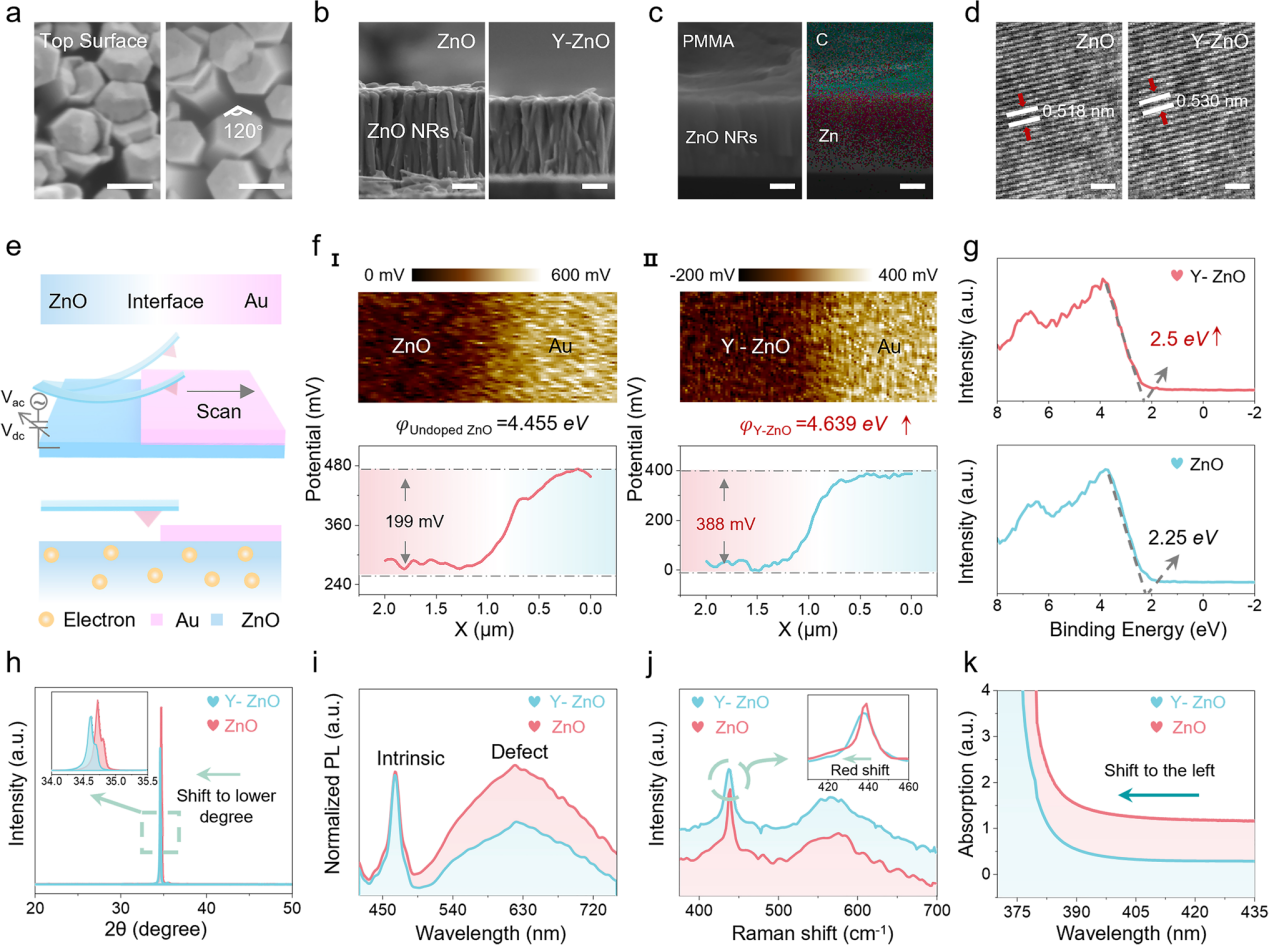
Microstructure and fundamental characterization of ZnO and Y-ZnO. **a** Cross-sectional and **b** top-view SEM images of undoped ZnO (left) and Y-ZnO (right) NRs. Scale bars, 100 nm. **c** Cross-sectional EDS spectra of the BPS. **d** HRTEM images of ZnO and Y-ZnO NRs. Scale bars, 0.5 nm. **e** Schematic illustration of the KPFM measuring surface potential. **f** Comparative surface potential profiles between undoped ZnO–Au (I) and Y-ZnO–Au (II). **g** VB-XPS, **h** XRD patterns, **i** room-temperature PL spectra, **j** Raman spectrum and **k** UV–vis absorption spectra of ZnO and Y-ZnO NRs

To construct high-performance piezotronic sensors, we systematically investigated the effects of Y-ions doping on piezoelectric output of ZnO. As shown in the crystal structure schematic (Fig. S1), Zn ions in the wurtzite ZnO lattice are partially replaced by Y-ions (Y-ZnO) to induce structural asymmetry [46–48]. Morphological characterization through SEM reveals well-aligned ZnO nanorods (NRs) with distinct hexagonal facets, demonstrating successful growth via the low-temperature hydrothermal method for both undoped (left) and Y-doped ZnO (right) samples (Fig. 2a, b). Elemental mapping analyses in Figs. 2c and S2, S3 confirm the uniform distribution of Zn, O, and C elements throughout the cross section, with Ag elements concentrated at the top surface, validating the successful preparation of the piezotronic sensor. X-ray photoelectron spectroscopy (XPS) analysis (Fig. S4) demonstrates the clear presence of Y elements in ZnO NRs, as evidenced by the distinct peaks corresponding to Y 3*d*_5/2_ (156.4 eV) and Y 3*d*_3/2_ (157.7 eV). High-resolution TEM (HRTEM) results reveal an expansion in crystalline interplanar spacing for Y-ZnO compared to undoped ZnO, implying an increase in lattice distortion (Fig. 2d). This structural modification significantly reduces free carrier concentration due to the enhanced carrier trapping by the distorted lattice. Furthermore, Kelvin probe force microscopy (KPFM) was employed to quantify the work function of ZnO before and after doping, as schematically illustrated in Fig. 2e. When a probe scanned from the ZnO surface to the Au surface, the surface potential of the sample can be calculated according to the equation:

1 $${V}_{\text{Au}-\text{Zn}0}=\frac{1}{e}\left({\varphi }_{\text{Zn}0}-{\varphi }_{\text{Au}}\right)$$

where ${V}_{\text{Au}-\text{Zn}0}$ is the difference in surface potential between Au and ZnO, *e* is the elementary charge, ${\varphi }_{\text{ZnO}}$ is the work function of ZnO, and ${\varphi }_{\text{Au}}$ is the work function of Au (5.1 eV). According to the KPFM scanning images (Fig. 2f), the work functions of undoped ZnO and Y-ZnO are 4.455 and 4.639 eV, respectively, corresponding to a 0.184 eV enhancement after doping. This finding is corroborated by valence band XPS (VB-XPS) results, which reveals a 0.25 eV increase in work function (Fig. 2g) [49, 50]. Meanwhile, structural characterization through XRD confirms the hexagonal wurtzite structure for both materials, with Y-ZnO showing a shift in the (0 0 2) diffraction peak to lower diffraction angles, suggesting expanded crystalline interplanar spacing after doping (Fig. 2h). In addition, the normalized room-temperature photoluminescence (PL) spectrum (under 325 nm continuous excitation) of the film demonstrates a reduction in oxygen vacancy (V_O_) related defects (at about 630 nm) with increasing doping concentration (Fig. 2i). At the same time, Raman spectroscopy reveals a redshift of the characteristic peak at 437 cm^−1^, confirming lattice modification (Fig. 2j). UV–vis absorption spectra (Fig. 2k) show a left shift with doping, indicating the band gap widening of the sample. As shown in Fig. S5, electrical characterization confirms n-type conductivity for both materials, with reduced carrier concentration in Y-ZnO. Figure S6 shows the change in the band gap of ZnO before and after doping [51], visually explaining the reason for the change in carrier behavior according to the following equation:

2 $${\left(\alpha hv\right)}^\frac{1}{n}={A}^{*}\left(hv-{E}_{\text{g}}\right)$$

where *α* is the absorption coefficient, *h* is Planck’s constant, *ν* represents the photon frequency, the slope of the tauc plot in the linear region is denoted by *A*^*∗*^ and *E*_g_ is the band gap. For direct band gap ZnO (*n* =1/2), the analysis yields a band gap of 3.23 eV (undoped) and 3.26 eV (Y-ZnO), respectively. Finally, finite element simulations show that a decrease in carrier concentration leads to an enhancement of the piezoelectricity, suggesting that doping is an effective strategy for modulating the performance of piezotronic sensors (Fig. S7).

### Electrical Performance of BPS

**Fig. 3:**
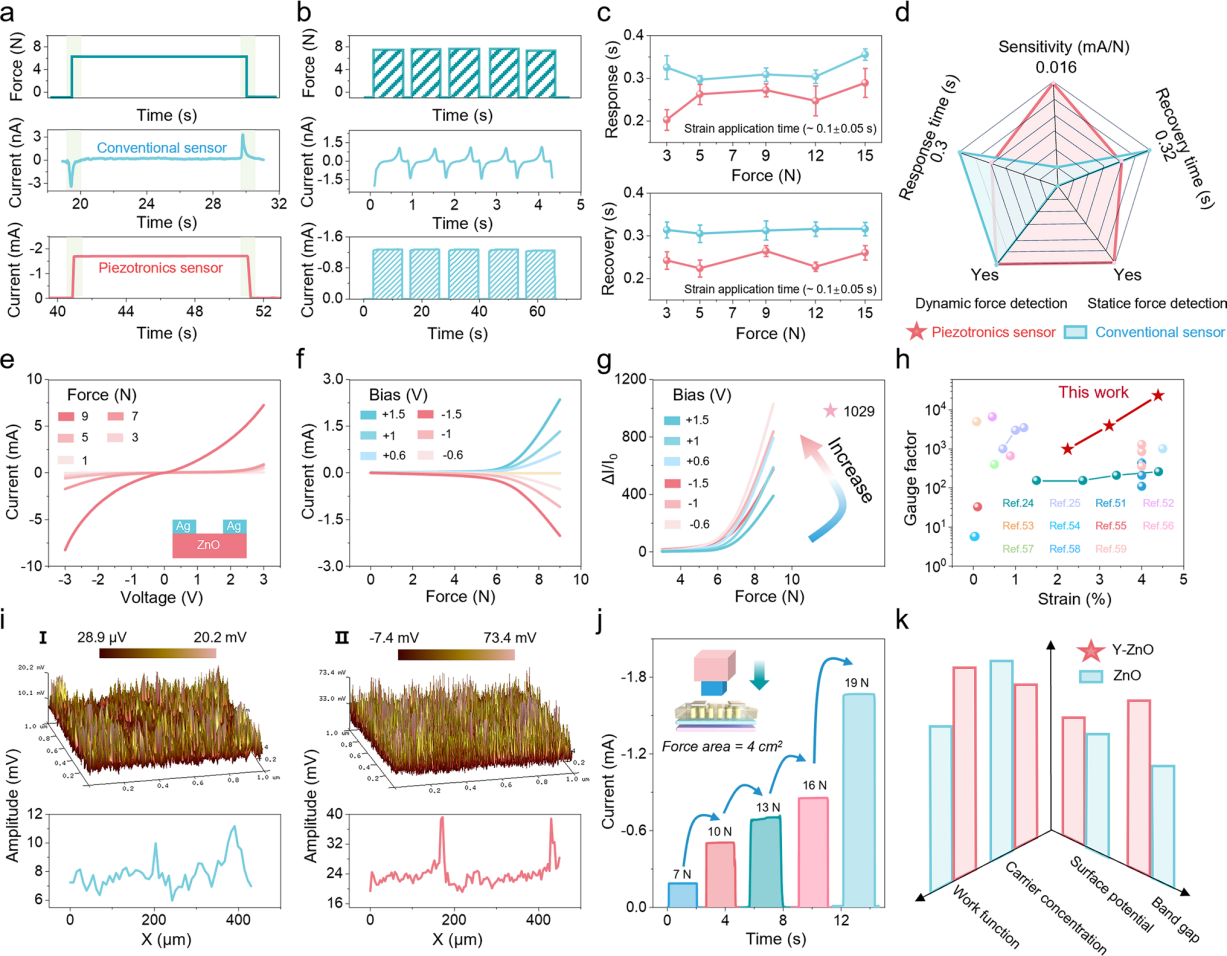
Piezotronic modulation and electrical characterization of piezotronic sensor. Comparison of conventional piezoelectric sensor and piezotronic sensor in **a** static force, **b** dynamic force detection and **c** response/recovery time. **d** Radar chart for comparing the different performances between the two sensors. **e** Pressure-dependent *I-V* characteristics of the BPS under the sweeping bias between − 3 and + 3 V. **f** Current-pressure relationship of the BPS under bias of − 1.5, − 1, − 0.6, 0, 0.6, 1, and 1.5 V. **g** Current change ratio $(\Delta I/I_{0} )$ as a function of pressure for the BPS under various forward and reverse bias conditions. **h** Comparison of gauge factor between this work and some existing ZnO-based sensors. **i** PFM testing results of ZnO and Y-ZnO. **j** Current response of the BPS under gradient static pressure of 7 ~ 19 N. **k** Fundamental property comparison between Y-ZnO and ZnO

The fundamental distinction between piezotronic sensors and conventional piezoelectric sensors lies in their capability to detect static forces. As shown in Fig. S8, conventional piezoelectric sensors exhibit transient electrical responses under constant force, whereas piezotronic sensors maintain stable output. By comparing their *I-T* curves under the same force, it can be seen that conventional piezoelectric sensors generate an electrical signal only at the instant when the force is applied or removed, whereas piezotronic sensors sustain stable output throughout the entire duration of force application (Fig. 3a). As shown in Fig. S9, the piezotronic sensor exhibits no significant signal degradation over 600 s of continuous force application. By increasing the frequency of the external force, the piezotronic sensor is able to track changes accurately, whereas the conventional piezoelectric sensor only generates transient responses (Fig. 3b). Based on the *I-T* curves, the average response time and recovery time of the BPS are shorter than that of the conventional piezoelectric device (Fig. 3c). A comprehensive performance comparison in Fig. 3d establishes the superiority of piezotronic sensors across all critical metrics. From the pressure-dependent *I-V* characteristics of the BPS (Fig. 3e), both forward and reverse currents change dramatically with the applied pressure, indicating the ability to respond to different external forces. In addition, a similar trend is observed across different bias conditions (Fig. 3f). Notably, the device achieves a remarkable current change ratio ( of 1029 at − 0.6 V bias under 9 N force (Fig. 3g), corresponding to 4.39% strain (Fig. S10), thus yielding an exceptional gauge factor of 23,439. Comparative analysis with some other existing ZnO-based piezotronic or conventional piezoelectric sensors highlights the superior performance of the BPS (Fig. 3h) [24, 25, 52–60], demonstrating its great potential for advanced strain sensing. As can be shown in Fig. S11, the piezoelectric output of the Y-ZnO is roughly fivefold higher than that of pure ZnO. The piezoelectric output remains at the same magnitude after exchanging positive and negative electrodes, excluding triboelectric interference (Fig. S12). Furthermore, the PFM images of ZnO and Y-ZnO, with randomly selected squares of 450 nm on each side, reveal enhanced piezoelectric output in Y-ZnO, with maximum potential fluctuations increasing from 20.2 to 73.4 mV after doping (Fig. 3i). Finally, we also measured the response of the BPS to a series of static pressures. As a result, the BPS demonstrates excellent static pressure response characteristics, accurately tracking both increasing and decreasing pressure profiles across multiple cycles (Fig. 3j). By comparing the relevant parameters of ZnO and Y-ZnO, it can be understood that the fundamental reason for the enhanced piezoelectric output is that doping reduces the carrier concentration, increases the band gap, and improves the work function and surface potential (Fig. 3k). The increased distance between the Fermi energy level and the conduction band reduces intrinsic electron concentration [61, 62], minimizing polarization charge screening and significantly enhancing piezoelectric response. As a result, these modifications facilitate carrier transport across the semiconductor interface, ultimately resulting in the improved performance of the BPS.

### Achilles Tendon Behavior Monitoring Assisted with Machine Learning

**Fig. 4:**
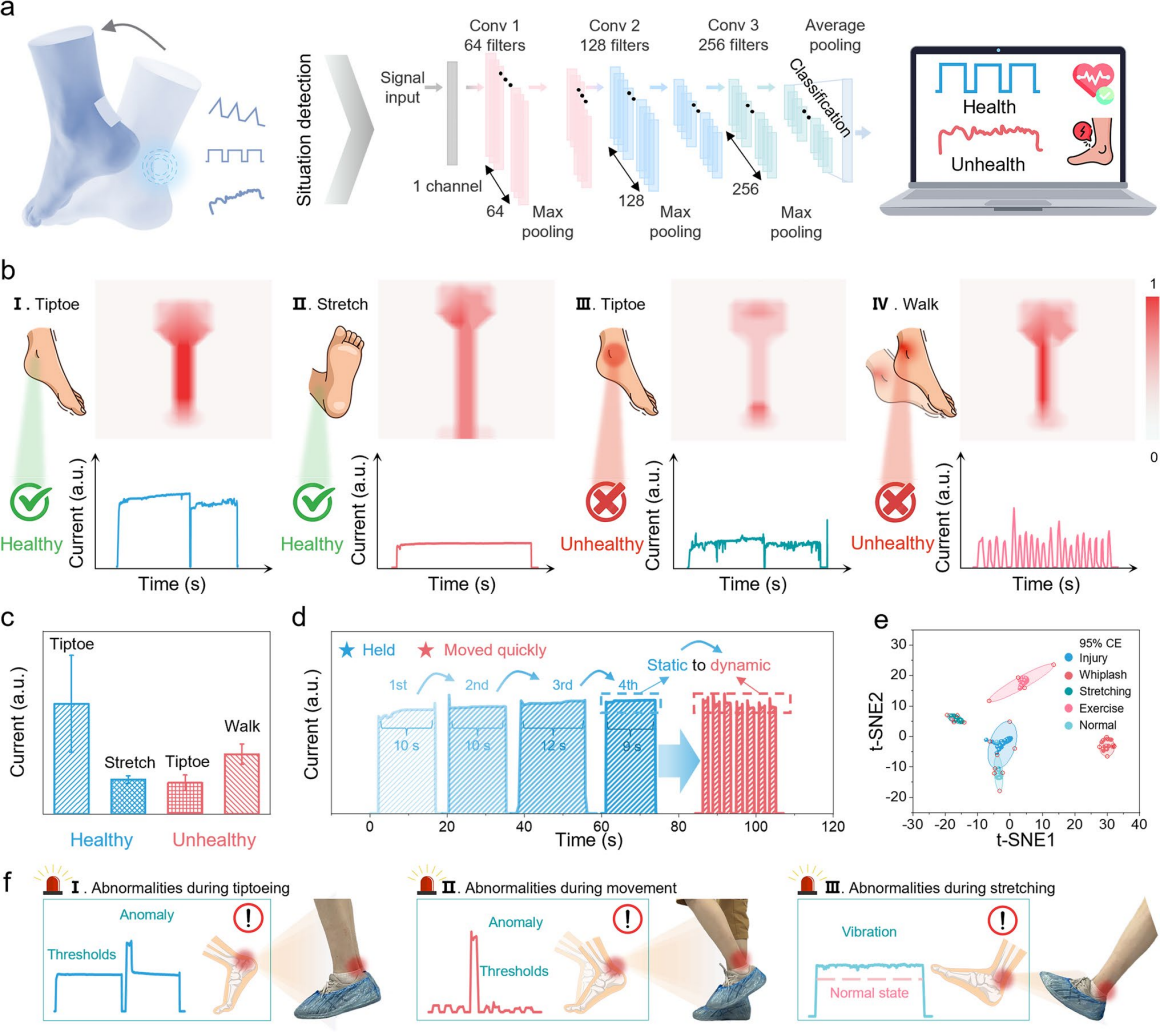
Achilles tendon behavior monitoring system based on BPS assisted with machine learning. **a** Schematic illustration of the BPS deployed on the Achilles tendon for capturing various postures, detailed architecture of the constructed 1D-CNN model for identifying health states. **b** Pressure distributions of 4 Achilles tendon behaviors and corresponding current responses. **c** Comparison of current intensity for 4 Achilles tendon behaviors. **d** Monitoring of dynamic and static forces during Achilles tendon rehabilitation exercises. **e** Visualizing the output data after deep learning adopting t-SNE dimensionality reduction. **f** Early warning during Achilles tendon movement

The Achilles tendon is particularly vulnerable to injury during overloaded conditions caused by improper exercise postures, such as overfatigue or excessive force [63]. Here, the developed BPS, combined with machine learning algorithms, enables Achilles tendon monitoring, as shown in Figs. 4a and S13. First, the BPS is attached to the Achilles tendon for movement state detection. Then, the obtained electrical signals are transmitted to the deep learning algorithms. Finally, the recognition results are displayed on the visualization interface for monitoring or early warning. In fact, different Achilles tendon states correspond to different stresses, resulting in differences in the magnitude and waveform of the outputs from the piezotronic sensors. In Fig. 4b, when performing a tiptoe stance with a healthy tendon, the BPS generates a strong and stable current output due to the uniform force distribution (I). Conversely, a heel stance results in reduced deformation and consequently weaker, but still stable, current output as the BPS changes from a bent state to a stretched state (II). When the Achilles tendon is in a disease state, the impaired muscle is unable to exert force properly, as evidenced by the weak current output during tiptoe stance, because the BPS has a small degree of bending (III). If the volunteer walks, the movement cannot be maintained normally due to the weakness and pain of the muscles, so the device shakes with the Achilles tendon, resulting in fluctuated and weak current output (IV). Figures 4c and S14 summarize the current intensities and waveform for the four states and the *I-V* characteristic curves for the normal and abnormal states, quantitatively comparing the electrical outputs for the different states. Finally, the capability of the BPS for continuous monitoring static and dynamic forces was validated through the Achilles tendon rehabilitation test (Fig. 4d). In the test, the volunteer stood on tiptoe, held it for 10 s, repeated it 4 times, and then moved quickly to relieve the muscle pain and discomfort. Clearly, the electrical output from the BPS matches the whole process perfectly, demonstrating its good motion detection capability. Furthermore, machine learning training was performed on the data to recognize different Achilles tendon states. With the assistance of the 1D-CNN algorithm, the proposed model achieves high classification accuracy and robustness after 100 training sessions (Fig. S15). The high-dimensional data of the output is transformed into the low-dimensional data via t-distributed stochastic neighbor embedding (t-SNE) visualization, which results in five distinct clusters with 95% confidence intervals, corresponding to different tendon states (Fig. 4e). The confusion matrix analysis demonstrates excellent recognition capability, with an overall classification accuracy of 96% (Fig. S16). Moreover, when the normal thresholds are set for the tiptoeing (Fig. 4f), exercising, and stretching, respectively, the system sends out a reminder to prevent the Achilles tendon from being damaged when the exercise is too strenuous. In summary, the above results demonstrate that the developed BPS represents a significant stride in simultaneous detection of dynamic and static forces, laying a solid foundation for its practical applications and contributes to the advancement of non-invasive monitoring technology.

## Conclusion

In summary, we demonstrated a high-sensitivity bimodal piezotronic sensor based on Y-ion-doped ZnO. Utilizing the piezotronic effect, the developed device exhibits exceptional electromechanical performance, with an on/off ratio of up to 1029, a gauge factor of up to 23,439, and sustained static force response capability exceeding 600 s. These characteristics represent significant advancements over conventional piezoelectric sensors, particularly in simultaneous static and dynamic force detection. As a proof-of-concept, the developed piezotronic sensor could accurately recognize the Achilles tendon behavior continuously, achieving classification accuracy of 96% for decoupling complex biomechanical signals. Excellent sensitivity and bimodal monitoring capability make the BPS ideal for user-friendly, long-term healthcare monitoring wearables. This work inspires dedication to developing promising bimodal sensors in digital health monitoring, intelligent soft robotic systems, and interactive wearable electronics.

## Supplementary Information

Below is the link to the electronic supplementary material.

Supplementary file1 (DOCX 4090 KB)
