# Supplementary material for: Piezotronic Sensor for Bimodal Monitoring of Achilles Tendon Behavior
Source: Nanomicro Lett. 2025 Apr 29;17:241. doi: 10.1007/s40820-025-01757-6 (PMC12040791; doi:10.1007/s40820-025-01757-6)
Supplement: Supplementary file 1 — Supplementary file1 (DOCX 4090 KB) [file 40820_2025_1757_MOESM1_ESM.docx]

Supporting Information for

**Piezotronic Sensor for Bimodal Monitoring of Achilles Tendon Behavior**

Zihan Wang^1^, Shenglong Wang^1^, Boling Lan ^1^, Yue Sun^1^, Longchao Huang ^1^, Yong Ao^1^, Xuelan Li^1^, Long Jin^1^, Weiqing Yang ^1,2^, Weili Deng^1,^ *

^1^Key Laboratory of Advanced Technologies of Materials (Ministry of Education), School of Materials Science and Engineering, Southwest Jiaotong University, Chengdu 610031, P. R. China

^2^Research Institute of Frontier Science, Southwest Jiaotong University, Chengdu 610031, P. R. China

*Corresponding author. E-mail: [weili1812@swjtu.edu.cn](mailto:weili1812@swjtu.edu.cn) (Weili Deng)

**Supplementary Figures**


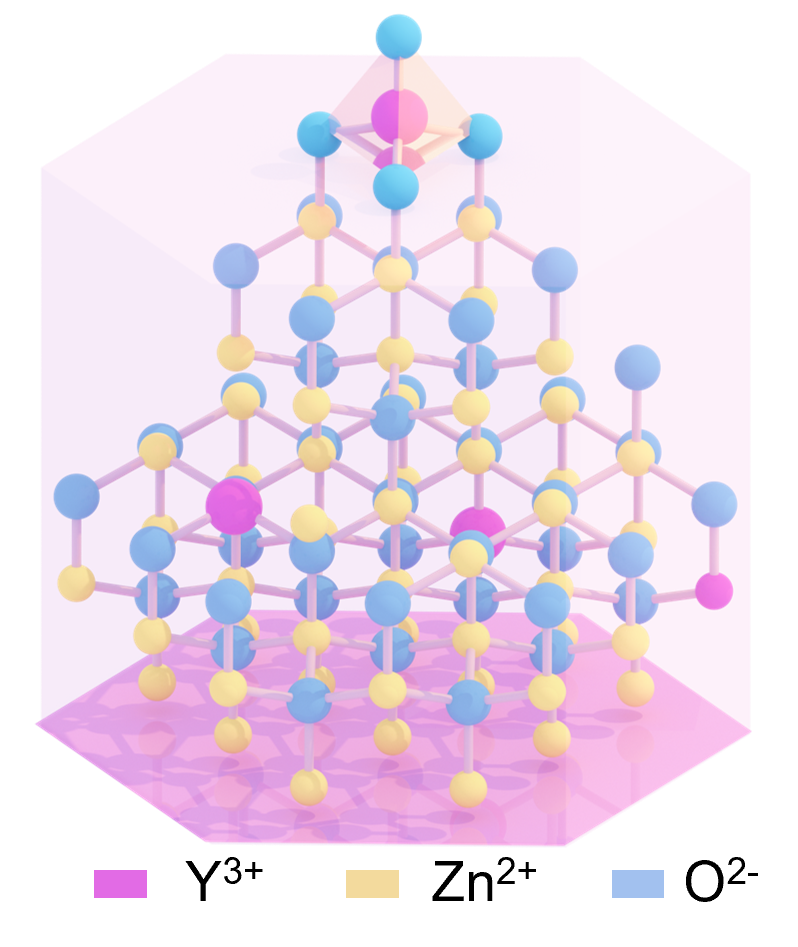


**Fig. S1** Schematic crystal structure of Y-doped ZnO NRs


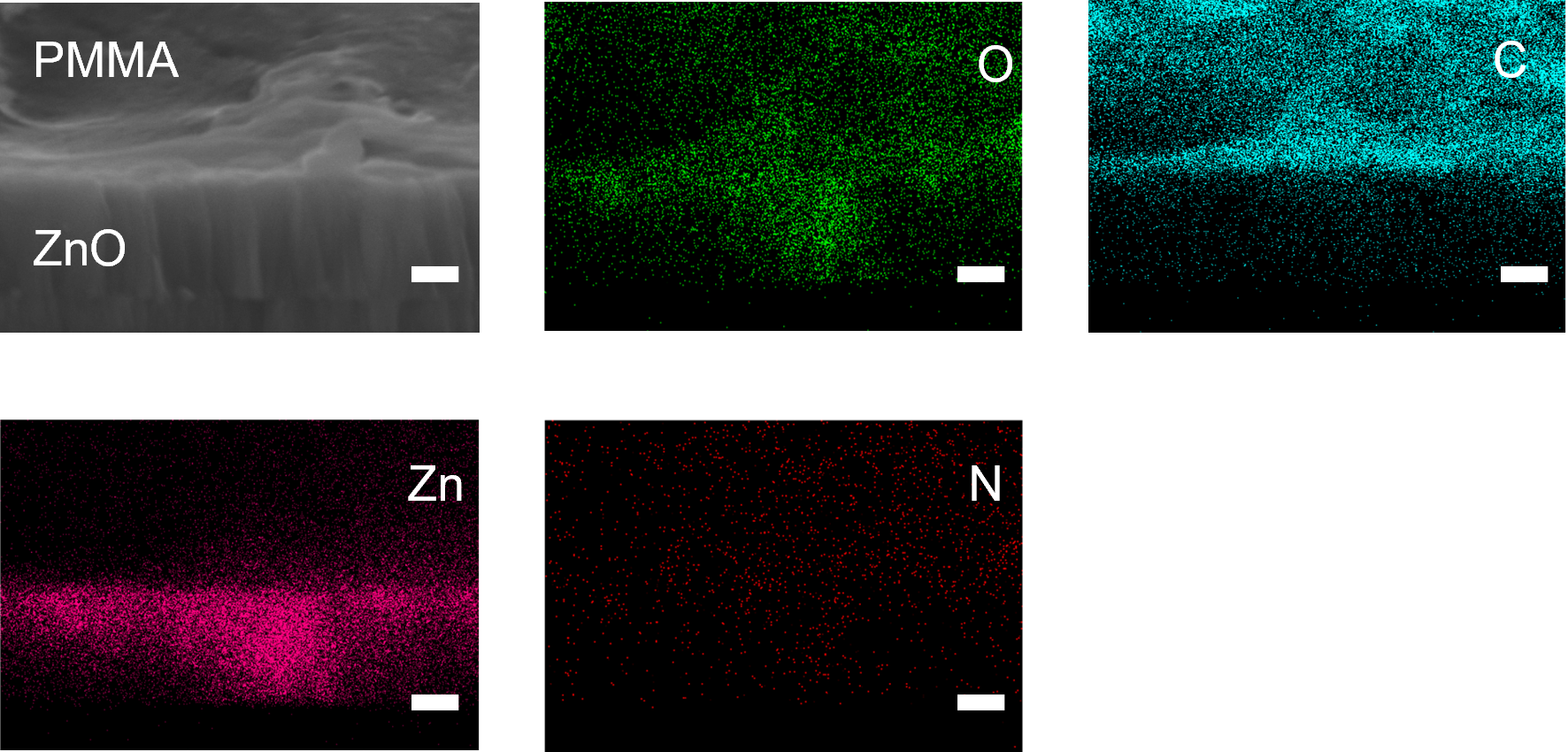


**Fig. S2** SEM image of the cross-sectional morphology and corresponding elemental [energy spectra](https://www.sciencedirect.com/topics/engineering/energy-spectra) of PMMA -ZnO. Scale bars, 500 nm


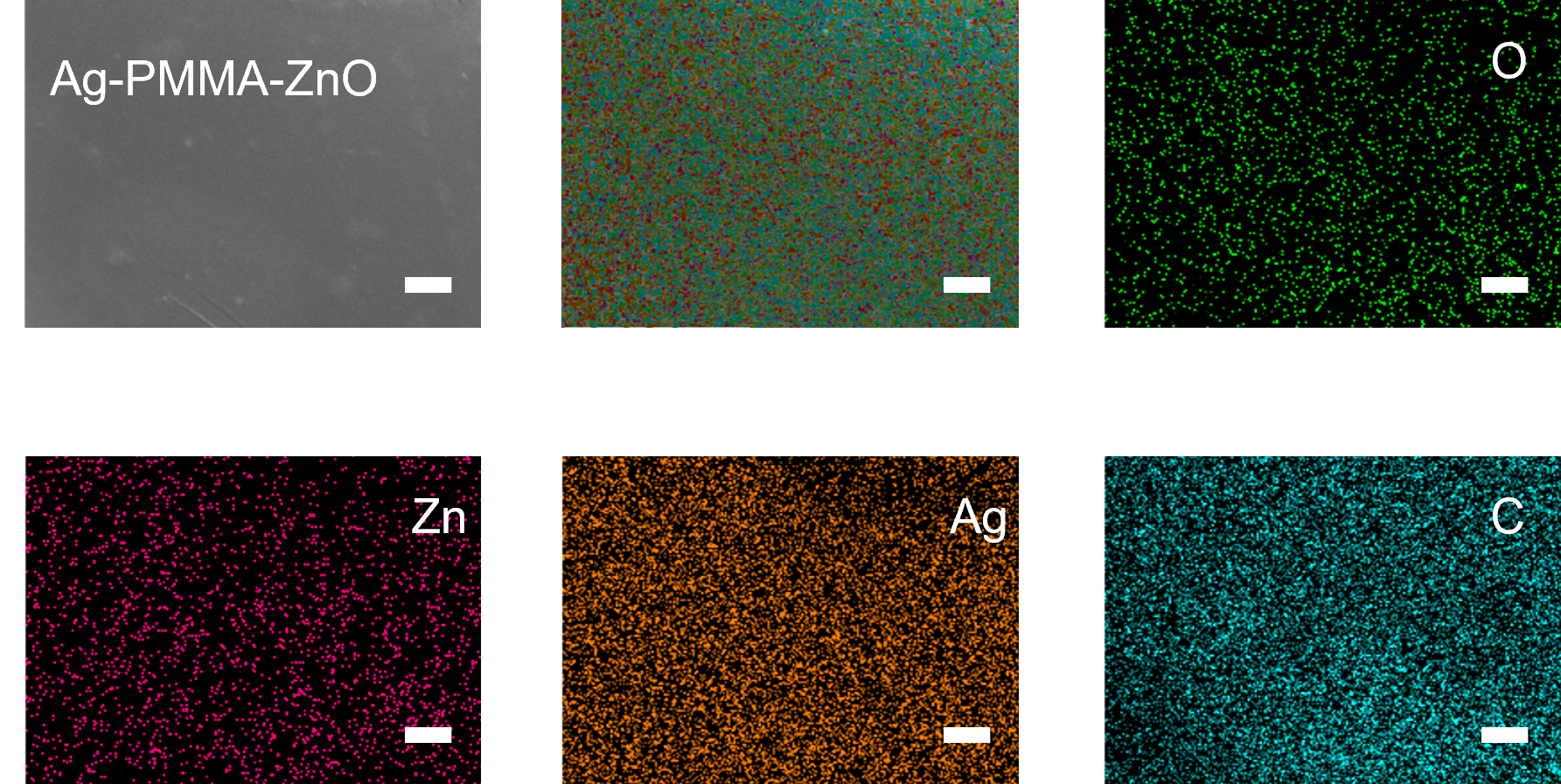


**Fig. S3** SEM image of the surface morphology and corresponding elemental [energy spectra](https://www.sciencedirect.com/topics/engineering/energy-spectra) of ZnO. Scale bars, 5 μm


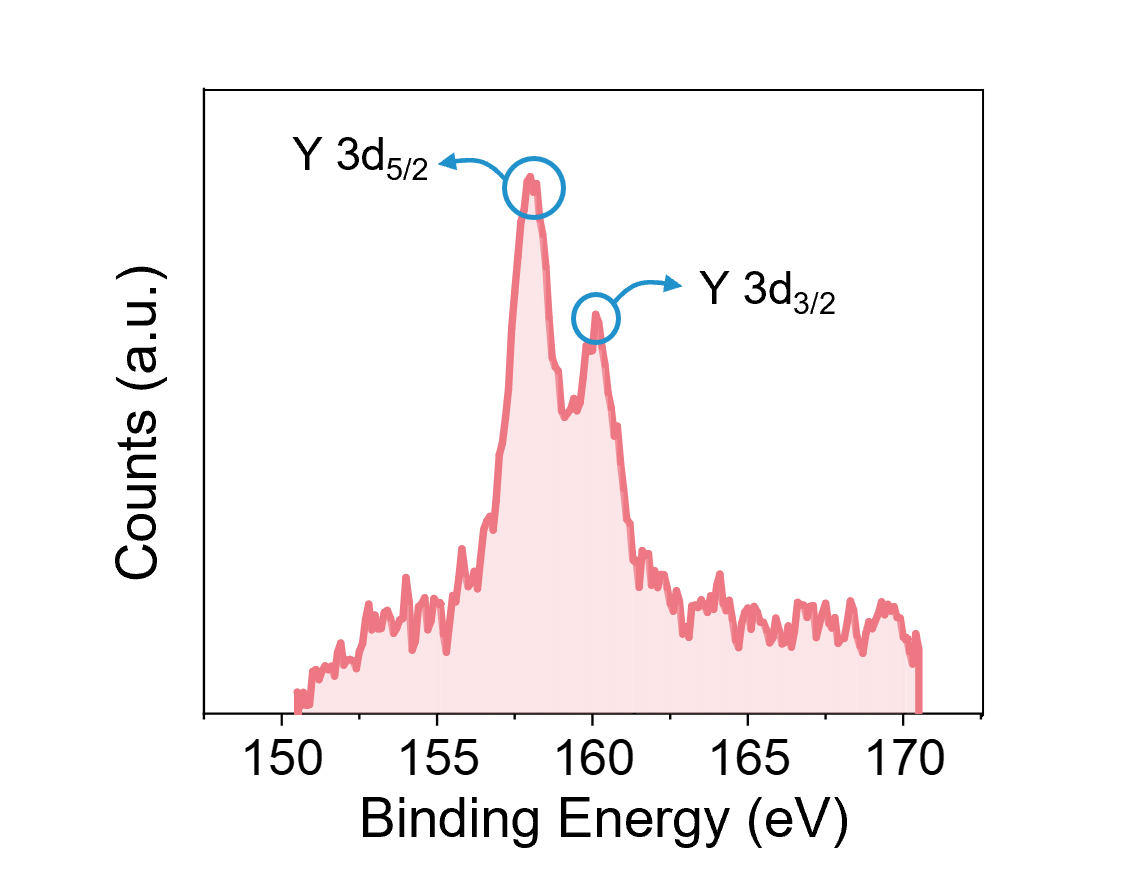


**Fig. S4** [XPS](https://www.sciencedirect.com/topics/materials-science/x-ray-photoelectron-spectroscopy) pattern of Y- ZnO NRs


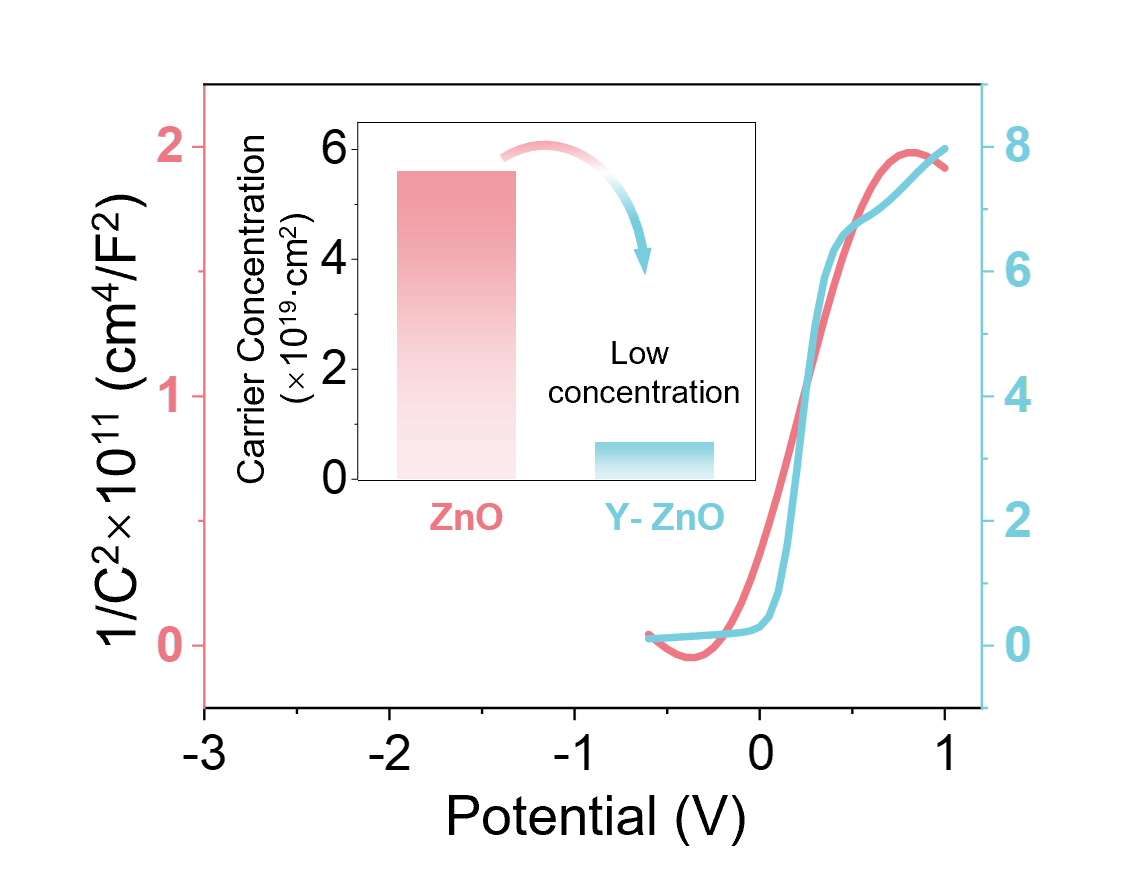


**Fig. S5** Mott-Schottky plots and linear fitting curves of ZnO NRs and Y-ZnO NRs


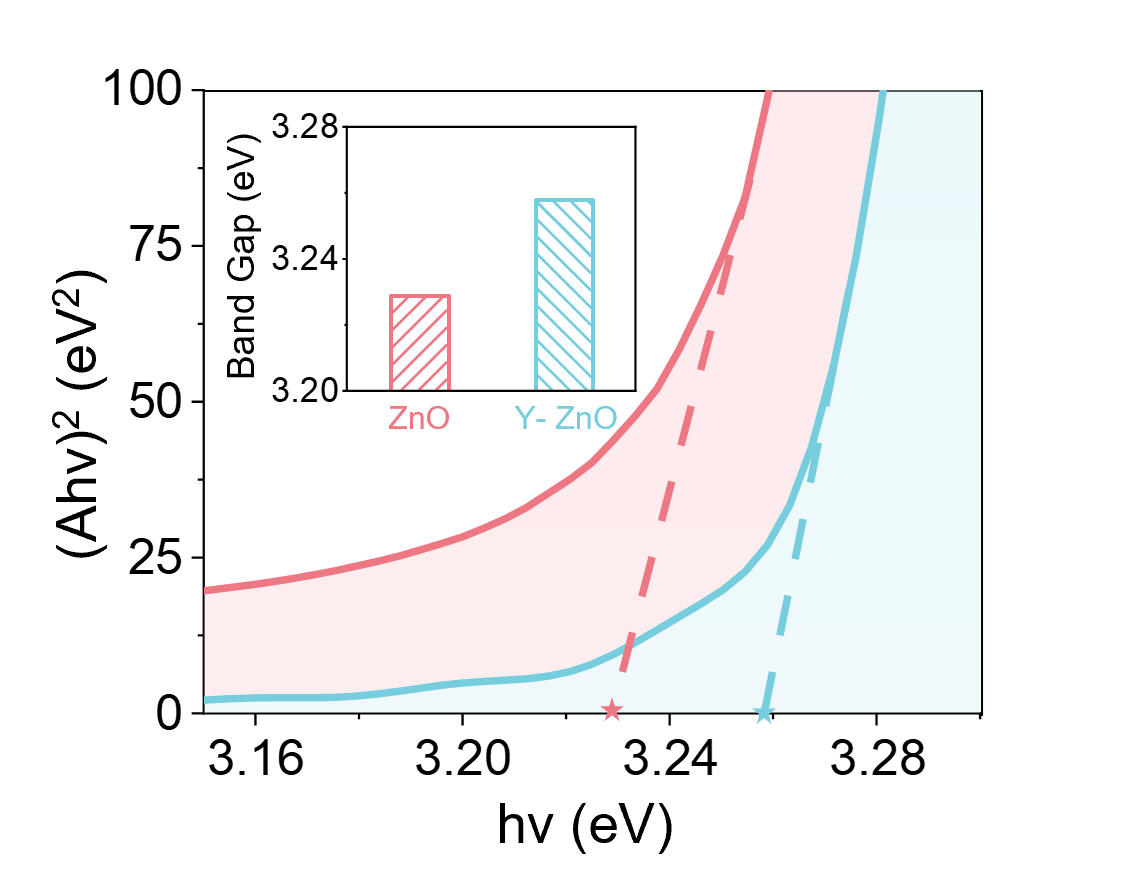


**Fig. S6** (*Ahν*) ^2^-*hν* graph calculated from [**Fig. 2**](https://www.sciencedirect.com/science/article/pii/S2211285522011028#fig0010)**k** and the band gap of ZnO and Y-ZnO calculated from (*Ahν*) ^2^-*hν* graph


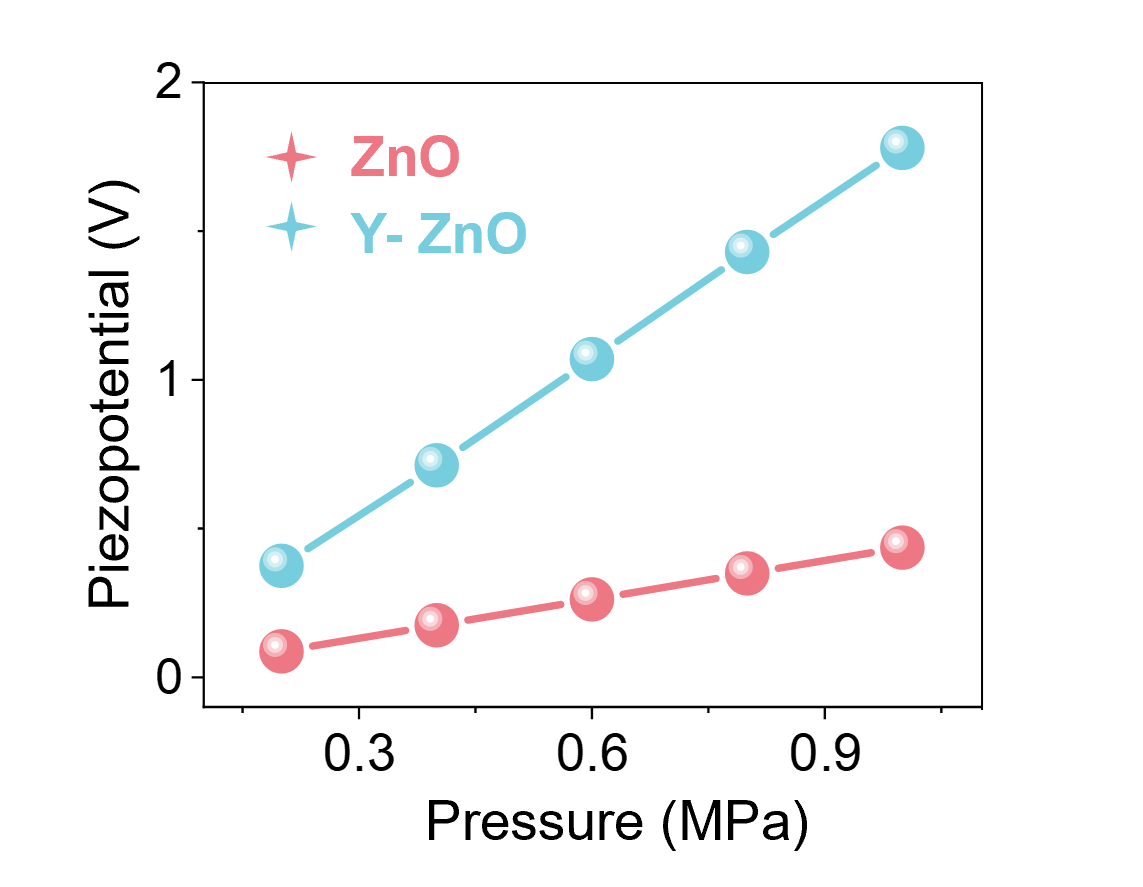


**Fig. S7** Simulation results of average sensitivities of ZnO and Y-ZnO piezoelectric sensor


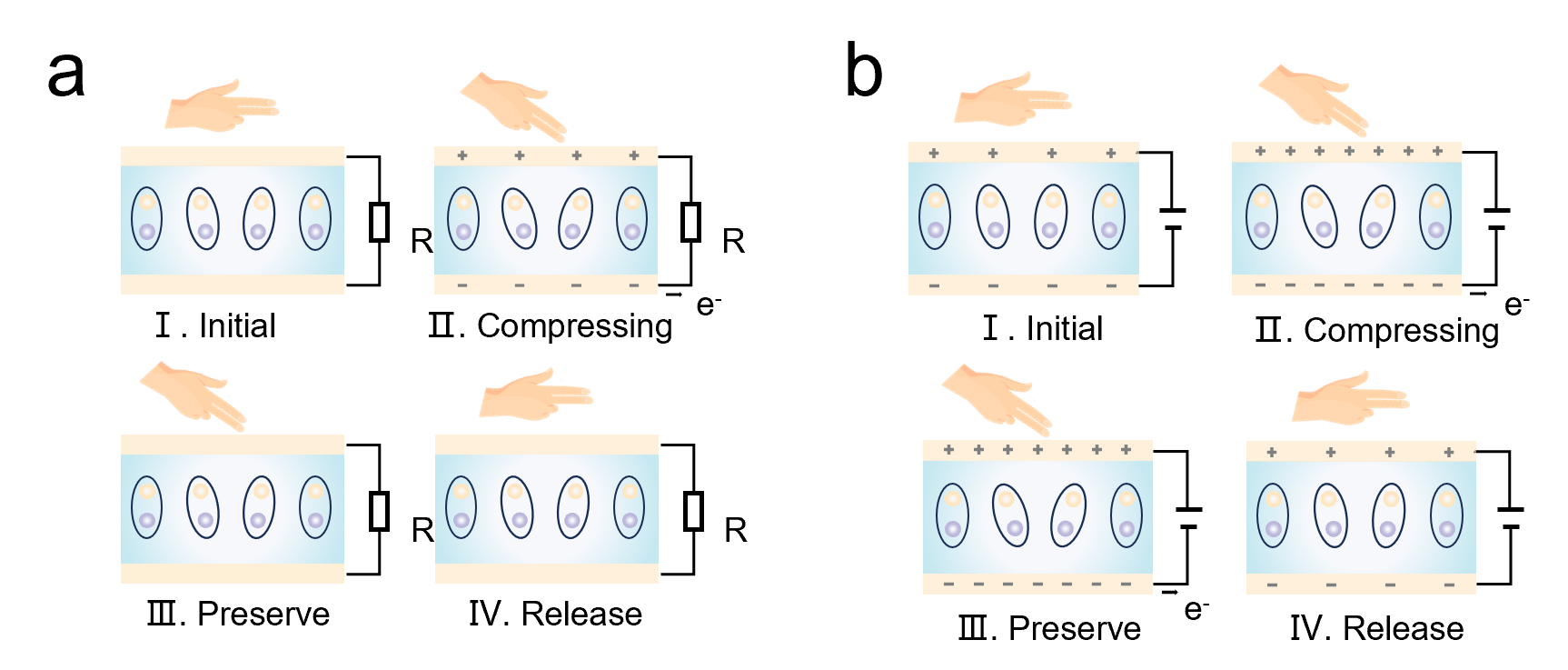


**Fig. S8** Schematic diagram of the working principle of conventional and piezotronic sensor. When stress is applied to a conventional piezoelectric device (**a**), there is a current passing through the external circuit for a split second due to the generation of a polarization charge, which disappears when the stress continues to be maintained and cannot be maintained with the external force. When stress is applied to a BPS (**b**), there is a change in the current due to a change because the polarization charge changes the interface barriers, and this change can be maintained under an external force until the external force is withdrawn


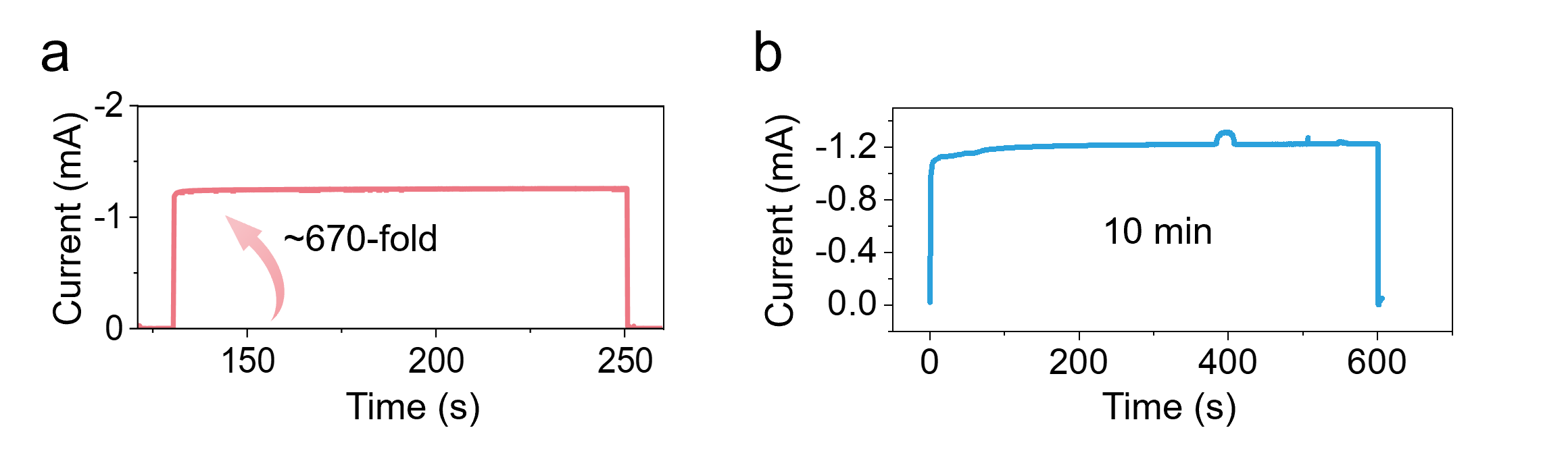


**Fig. S9** *I-T* curve of BPS held at the force of 5 N with 120 s (**a**) and 600 s (**b**)

**
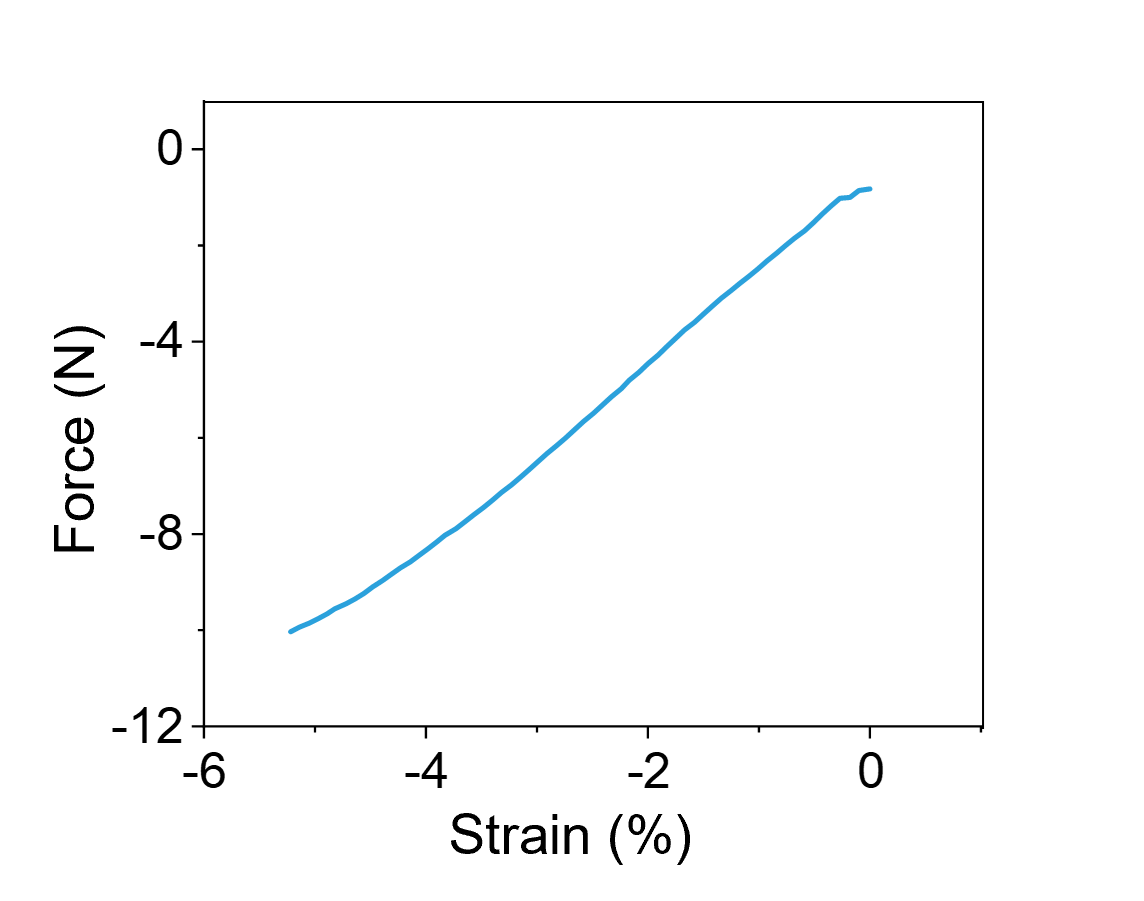
**

**Fig. S10** Corresponding curve of stress and strain

**
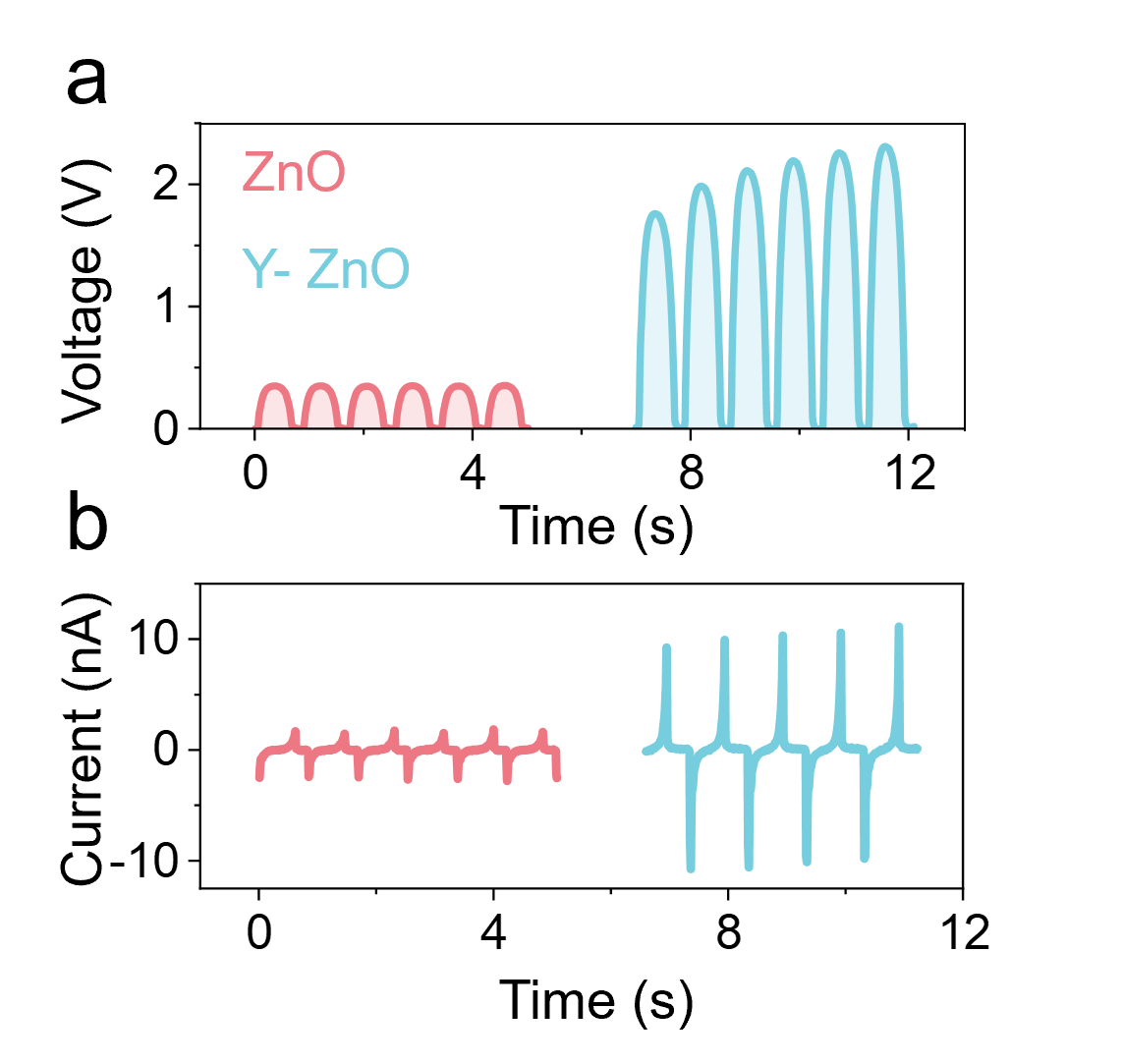
**

**Fig. S11** Voltage output (**a**) and current output (**b**) of ZnO and Y-ZnO under the same pressure

**
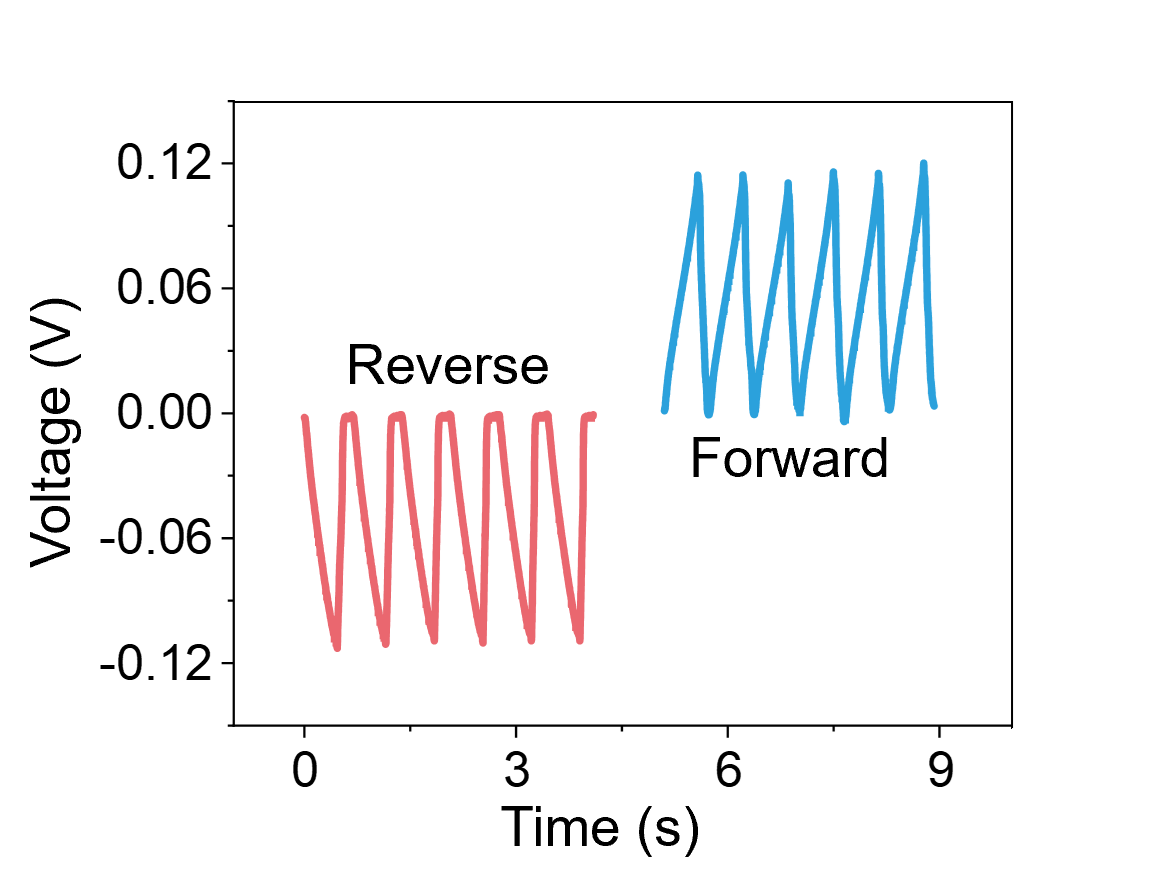
**

**Fig. S12** Polarity switching test of ZnO piezoelectric sensors

**
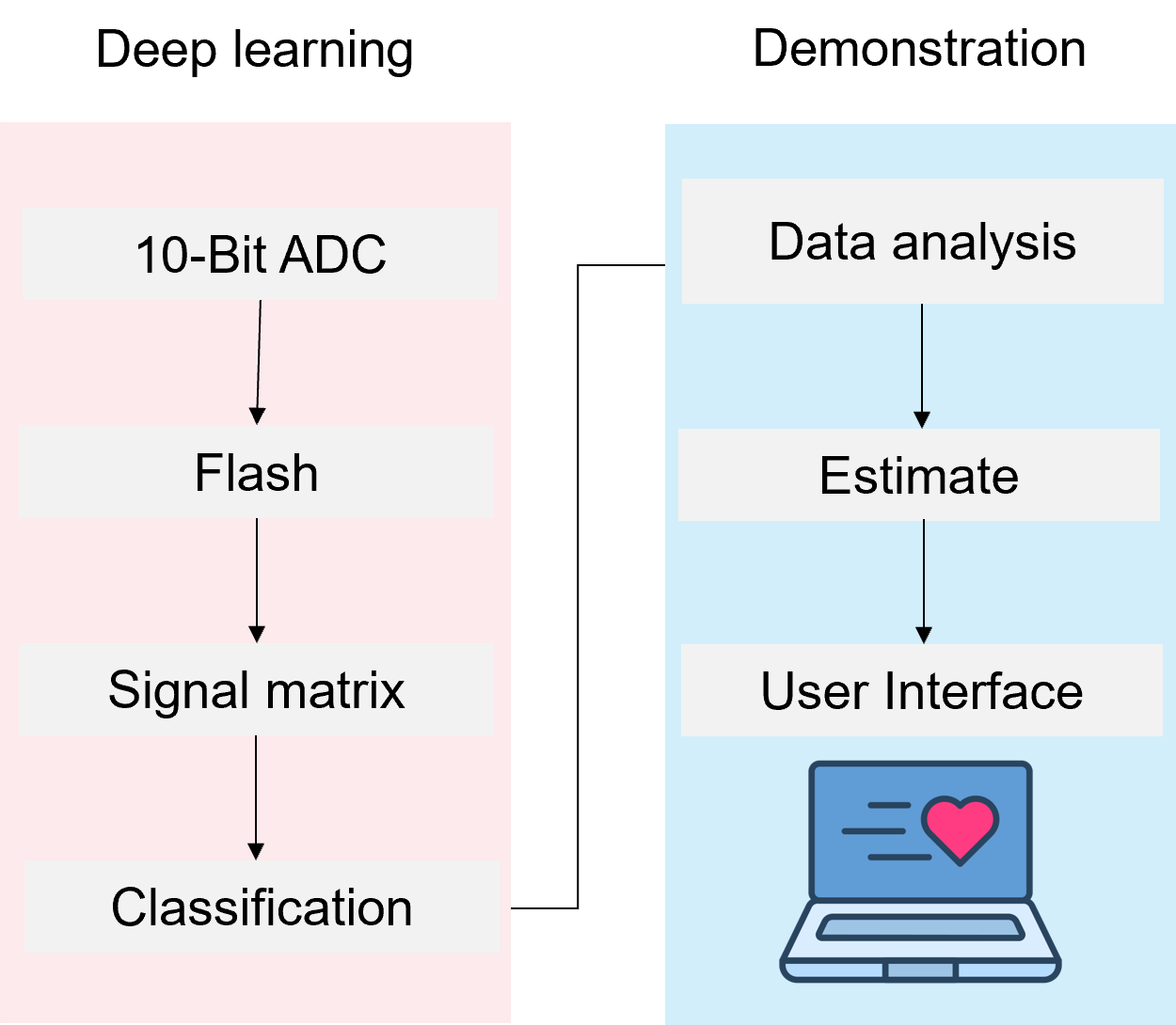
**

**Fig. S13** Schematic illustration of the deep-learning-assisted human motion capture system

**
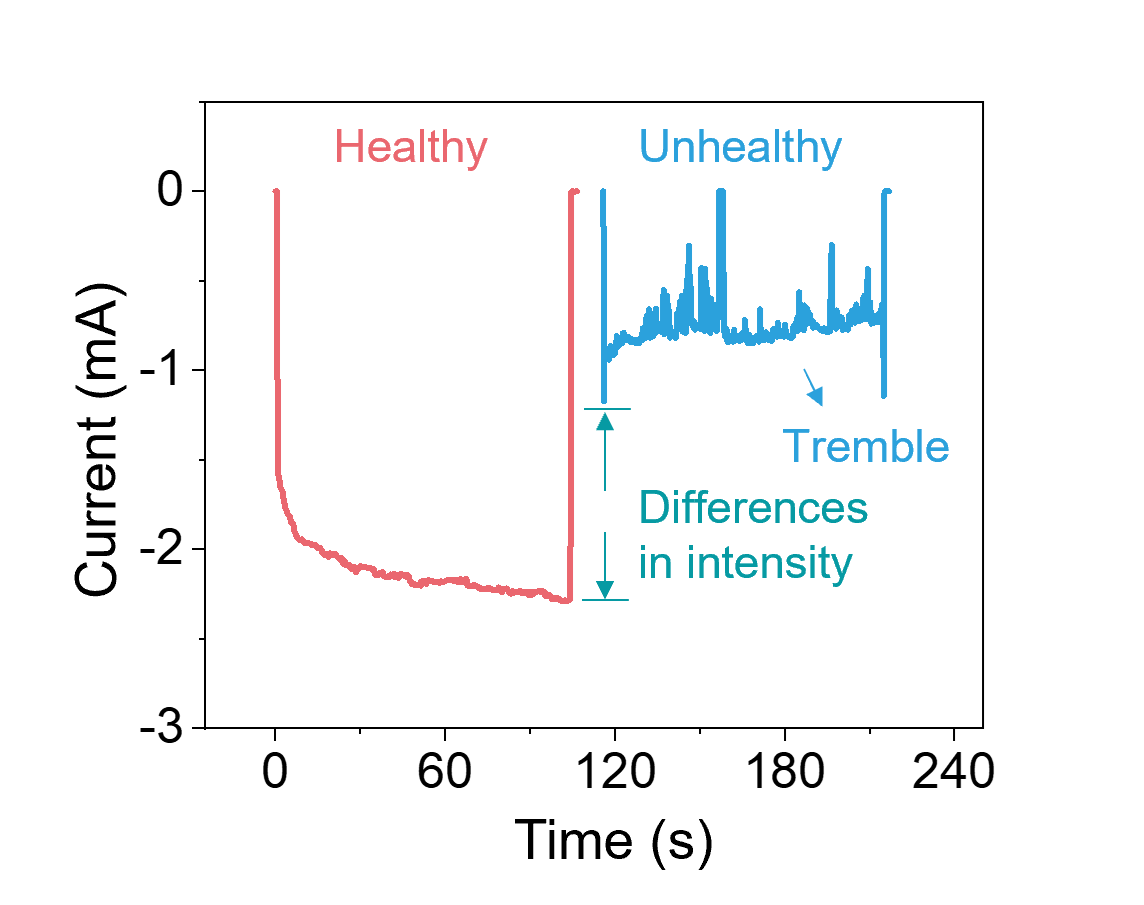
**

**Fig. S14** Comparison of characteristic current curves of BPS in healthy and unhealthy states

**
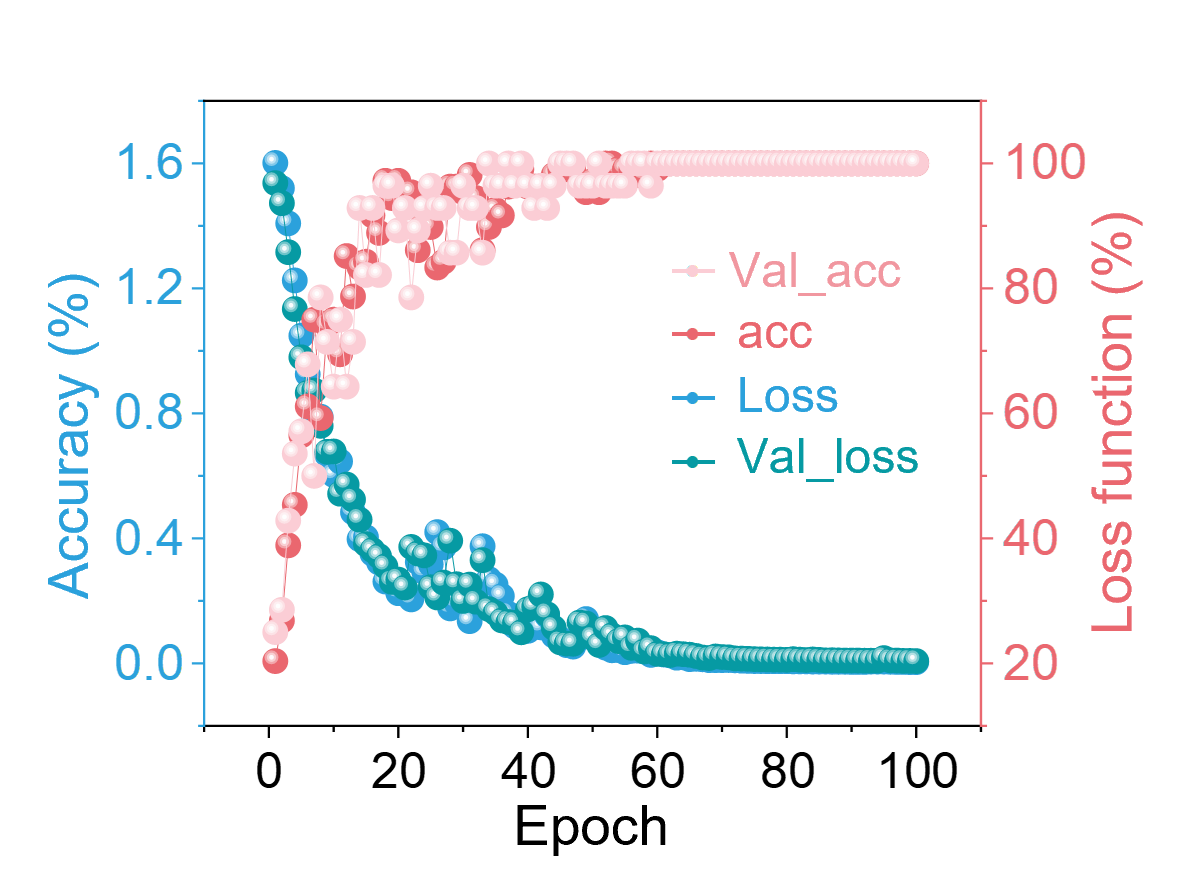
**

**Fig. S15** Classification accuracy and loss function of training and validation datasets in 100 epochs


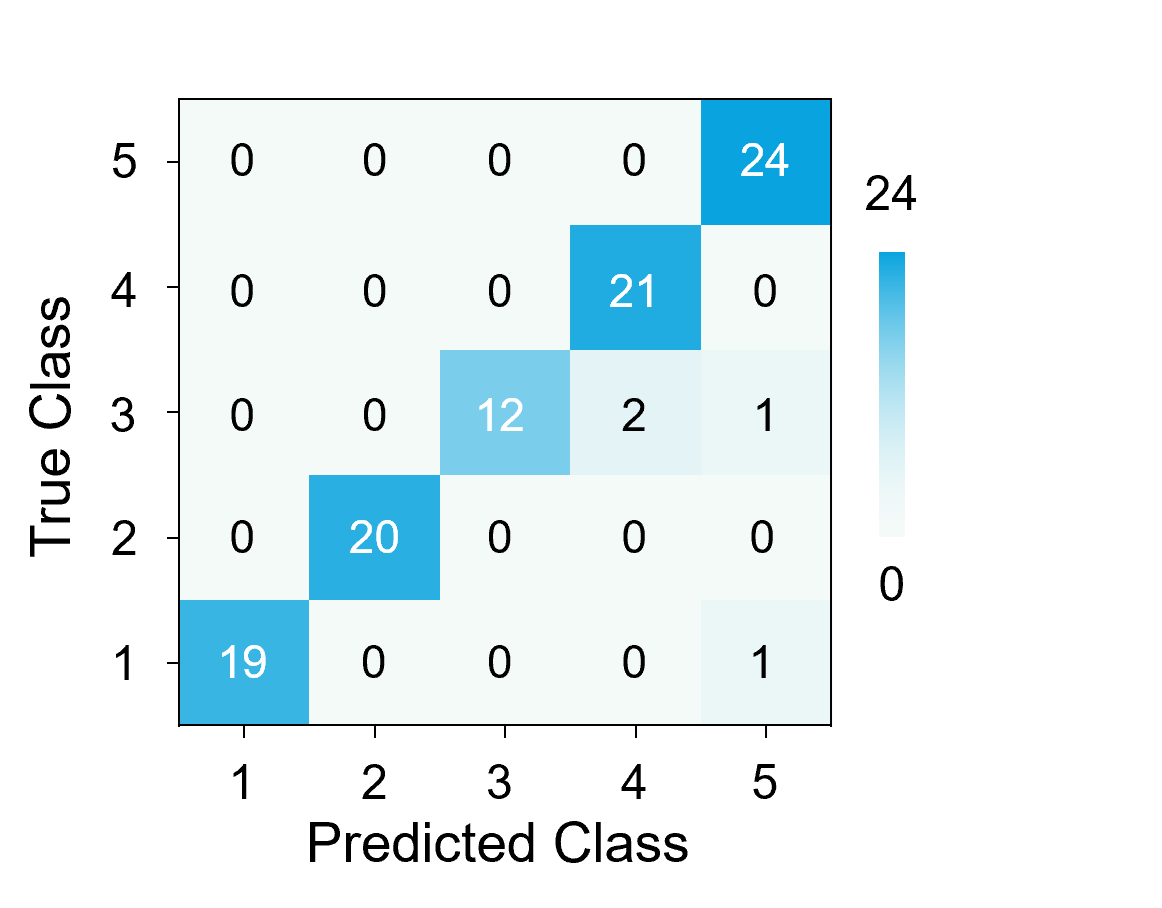


**Fig. S16** Confusion matrix for the recognition of 5 Achilles tendon behaviors
